# Predictors of Dermal Invasion and Sentinel Lymph Node Metastasis in Patients With Extramammary Paget's Disease Without Clinical Lymph Node Enlargement

**DOI:** 10.1111/1346-8138.17795

**Published:** 2025-05-23

**Authors:** Ken Horisaki, Shusuke Yoshikawa, Wataru Omata, Arata Tsutsumida, Yoshio Kiyohara

**Affiliations:** ^1^ Department of Dermatology Shizuoka Cancer Center Shizuoka Japan; ^2^ Department of Dermatology Nagoya University Graduate School of Medicine Nagoya Japan

**Keywords:** extramammary Paget's disease, metastasis, prognosis, sentinel lymph node, sentinel lymph node biopsy

## Abstract

Sentinel lymph node biopsy (SLNB) has prognostic value in extramammary Paget's disease (EMPD) without lymph node enlargement; however, its therapeutic value is unknown. The likelihood of sentinel lymph node metastasis is extremely low, especially when the primary tumor is an intraepidermal lesion; therefore, sentinel node biopsy should not be performed in such cases. To avoid excessive sentinel node biopsy in patients with EMPD, we investigated the preoperative biomarkers that predict the degree of invasiveness of the primary tumor and sentinel lymph node metastasis. We reviewed 121 patients who underwent primary resection and SLNB. The invasion level of the primary tumor was intraepidermal in 50 patients (41.3%), microinvasion into the papillary dermis in 34 (28.1%), and deep invasion into/beyond the reticular dermis in 37 (30.6%). The sentinel node metastasis was positive in 0%, 5.9%, and 62.2% of patients in the intraepidermal, microinvasion, and deep invasion groups, respectively. Presence of nodules (odds ratio: 6.820, *p* = 0.001) and neutrophil‐to‐lymphocyte ratio (NLR) (≥ 3.03, odds ratio: 4.260, *p* = 0.009) were identified as independent predictive factors for deep dermal invasion of the primary tumor and sentinel node metastasis (with nodules, odds ratio: 8.460, *p* < 0.001 and NLR ≥ 2.87, odds ratio: 3.870, *p* = 0.016). The metastasis‐positive group had a significantly lower overall survival than the negative group (median overall survival: 27.6 months vs. not reached, log‐rank test, *p* < 0.001). In conclusion, routine SLNB may be useful for predicting the prognosis of patients with EMPD without clinical lymph node enlargement. However, the likelihood of sentinel lymph node metastasis is extremely low in intraepidermal and microinvasive primary lesions. It may be reasonable to proactively recommend SLNB, particularly in cases with confirmed deep invasion lesions, the presence of nodules, or elevated NLR.

## 1 Introduction

Extramammary Paget's disease (EMPD) is a rare cutaneous malignancy that mainly affects the genitalia, perianal area, and axillae in older adults. Generally, EMPD progresses slowly, and many patients with EMPD have no clinically enlarged lymph nodes. In such cases, in addition to primary tumor resection, sentinel lymph node biopsy (SLNB) is an option to check for potential lymph node metastases. However, there is little evidence regarding whether SLNB should be performed in patients with EMPD without clinical lymph node enlargement from a therapeutic aspect. There is also little evidence that additional treatments, such as adjuvant therapy or lymph node dissection, improve disease‐specific survival in the presence of sentinel lymph node (SLN) metastasis, and the Western guideline for EMPD does not recommend the routine use of SLNB [1]. However, the Japanese guidelines for EMPD weakly recommend performing SLNB in EMPD without clinical lymph node enlargement when dermal invasion of the primary tumor is suspected [2], since SLNB is a relatively safe procedure and the presence of lymph node metastasis can be a prognostic factor. However, in practice, it is often difficult to determine whether dermal invasion has occurred in the primary tumor without resecting the entire primary lesion. Therefore, even if a prior partial biopsy detects an intraepidermal (IE) EMPD lesion, primary tumor resection and SLNB are often performed simultaneously because of the patient burden of performing these two surgeries separately. Kibbi et al. reported that 21.7% (137 of 630) of patients with EMPD who underwent SLNB were positive for SLN metastasis; conversely, approximately 80% of patients were SLN metastasis‐negative and may have undergone excessive SLNB [1]. Several studies reported no positive SLN metastasis if the primary site of EMPD was an IE lesion [3, 4, 5]. Therefore, SLNB is overtreated in EMPD without clinical lymphadenopathy when the primary tumor is an IE lesion. However, few studies have investigated the preoperative biomarkers that predict dermal invasion of the primary tumor and SLN metastasis in patients with EMPD limited to the genital area. Therefore, in this study, we investigated whether the clinical characteristics and biomarkers prior to primary tumor resection could predict the invasion level of primary tumors and SLN metastasis.

## 2 Methods

### 2.1 Study Population and Data Collection

Patients who underwent primary tumor resection and SLNB for EMPD in the genital region between August 2002 and December 2024 were retrospectively reviewed. Clinical data such as patient age, sex, location, size, and invasion level of the primary tumor, presence or absence of ulcers and nodules, time from appearance of symptoms related to EMPD to surgery, and preoperative neutrophil‐to‐lymphocyte ratio (NLR) were collected. The invasion level of the primary site was classified as IE, microinvasion into the papillary dermis (MI), or deep invasion into/beyond the reticular dermis (DI). Ulcers and nodules at the primary site included those that were clinically but not necessarily pathologically determined. Ulcers also included erosions, and nodules were defined as having a height of ≥ 2 mm and a long diameter of ≥ 10 mm. Symptoms associated with EMPD include persistent itching, pain, and erythema of the genital area. Patients with primary EMPD other than in the genital area, such as the axilla and umbilicus, and those with secondary EMPD of anal origin were excluded.

### 2.2 Statistical Analysis

Baseline characteristics were compared using the Mann–Whitney *U* test for continuous variables and the chi‐square test for categorical variables. Receiver operating characteristic (ROC) curves were constructed using the DeLong model to determine the optimal NLR cutoff value. The associations of clinical factors with DI of the primary lesion and SLN metastasis were determined using multiple logistic regression models. Overall survival (OS) in various groups was estimated using the Kaplan–Meier method and evaluated using the log‐rank test. OS was defined as the time from surgery to the last follow‐up or death from any cause. OS was compared according to the invasion level at the primary site and SLN metastasis. As a subgroup analysis, OS was also compared by SLN metastasis in the MI and DI groups only, except for IE lesions. Statistical significance was set at *p* < 0.05. All analyses were performed using EZR version 1.55 for Windows OS XP~11.

### 2.3 Ethics Statement

This retrospective cohort study was conducted at Shizuoka Cancer Center, Shizuoka, Japan, and was approved by the Institutional Review Board of our hospital (approval number: J2024‐234). All personal data were handled in strict accordance with the ethical guidelines of the 1964 Declaration of Helsinki.

## 3 Results

### 3.1 Patient Characteristics Grouped by the Invasion Level of the Primary Lesion

A total of 121 patients underwent resection of the primary lesion and SLNB, and patient characteristics according to the invasion level of the primary lesion are summarized in Table 1. There was no difference in age between the three groups (*p* = 0.263). Regarding sex, males were significantly affected (*p* = 0.006). There was no difference in the frequency of the primary site extending into mucosal areas, such as the labia minora, clitoris, and urethra (*p* = 0.061). The deeper the lesion invasion, the more ulcers (*p* = 0.072) and nodules (*p* < 0.001) tended to be found in the primary lesions. In addition, the deeper the lesions invaded, the higher the preoperative NLR tended to be (*p* = 0.010). There were no differences in the time from symptom onset to surgery (*p* = 0.355), primary lesion length (mm) (*p* = 0.397), or product of the long and short diameters (cm^2^) (*p* = 0.592) among the three groups. Additionally, 83 patients were diagnosed with IE lesions based on preoperative partial biopsy, of which 50 (60.2%) had true IE lesions, 12 (14.5%) had MI, and 21 (25.3%) had DI.

**TABLE 1:** Characteristics of patients with EMPD who underwent primary tumor resection and SLNB.

Characteristic	Patient group (%)				*p*
	Total	IE	MI	DI	
Patients, *n* (%)	121 (100)	50 (41.3)	34 (28.1)	37 (30.6)	
Age, years					0.263
Median [range]	71 [36, 94]	70 [47, 94]	72 [36, 91]	74 [48, 94]	
Sex, *n* (%)					**0.006** *
Male	70 (57.9)	21 (42.0)	21 (61.8)	28 (75.7)	
Female	51 (42.1)	29 (58.0)	13 (38.2)	9 (24.3)	
Duration from symptoms to surgery (year), *n* (%)					0.355
≤ 1 year	49 (40.5)	18 (36.0)	18 (52.9)	13 (35.1)	
1–3 years	45 (37.2)	18 (36.0)	10 (29.4)	17 (45.9)	
≥ 3 years	27 (22.3)	14 (28.0)	6 (17.6)	7 (18.9)	
Presence of mucosal lesions, *n* (%)					0.061
Yes	39 (32.2)	22 (44.0)	9 (26.5)	8 (21.6)	
No	82 (67.8)	28 (56.0)	25 (73.5)	29 (78.4)	
Presence of ulcer, *n* (%)					0.072
Yes	84 (69.4)	31 (62.0)	22 (64.7)	31 (83.8)	
No	37 (30.6)	19 (38.0)	12 (35.3)	6 (16.2)	
Presence of nodules, *n* (%)					**< 0.001** *
Yes	23 (19.0)	3 (6.0)	3 (8.8)	17 (45.9)	
No	98 (81.0)	47 (94.0)	31 (91.2)	20 (54.1)	
Long diameter of the lesion (mm)					0.397
Median [range]	80 [25, 250]	90 [30, 230]	80 [40, 170]	70 [25, 250]	
Product of the long and short diameters of the lesion (cm^2^)					0.592
Median [range]	49.0 [6.3, 1356.8]	55.0 [6.8, 1356.8]	46.8 [12.0, 153.0]	38.5 [6.3, 625.0]	
NLR					**0.010** *
Median [range]	1.93 [0.61, 8.90]	1.82 [0.61, 6.4]	2.02 [0.83, 4.03]	2.61 [1.07, 8.9]	

Abbreviations: DI, deep invasion into/beyond the reticular dermis; EMPD, extramammary Paget's disease; IE, intraepidermal; MI, microinvasion into the papillary dermis; NLR, neutrophil‐to‐lymphocyte ratio; SLN, sentinel lymph node; SLNB, sentinel lymph node biopsy.

* Bold letters indicate stastically significant differences: *p* < 0.05.

### 3.2 Evaluation of SLN Metastasis by the Invasion Level of the Primary Lesion in EMPD

Of the 121 patients with EMPD, 20.7% had SLN metastasis (Table 2). All 121 patients underwent SLNB at the same time as the primary resection. The positivity rate of SLN metastasis increased significantly with deeper tumor invasion of the primary lesion (*p* < 0.001).

**TABLE 2:** Evaluation of SLN metastasis by the invasion level of the primary lesion in EMPD.

	Patient group (%)				*p*
	Total *n* = 121 (100)	IE *n* = 50 (41.3)	MI *n* = 34 (28.1)	DI *n* = 37 (30.6)	
Positive SLN metastasis, *n* (%)	25 (19.7)	0 (0.0)	2 (5.9)	23 (62.2)	**< 0.001** *
Negative SLN metastasis, *n* (%)	96 (75.6)	50 (100)	32 (94.1)	14 (37.8)	

Abbreviations: DI, deep invasion into/beyond the reticular dermis; EMPD, extramammary Paget's disease; IE, intra‐epidermis; MI, microinvasion into the papillary dermis; SLN, sentinel lymph node.

* Bold letters indicate stastically significant differences: *p* < 0.05.

### 3.3 Univariate and Multivariate Analyses of Potential Predictors of DI

Multiple logistic regression analysis results showing the potential predictors of DI of the primary lesion are shown in Table 3. The most sensitive and specific cutoff value for NLR to predict DI of the primary lesion was determined to be 3.03 using the ROC curve.

**TABLE 3:** Multiple logistic regression analysis of predictors of deep invasion into/beyond the reticular dermis of patients with EMPD.

Variable	Univariate analysis			Multivariate analysis		
	Odds ratio	95% CI	*p*	Odds ratio	95% CI	*p*
Sex						
Female	Reference			Reference		
Male	3.110	1.310–7.380	**0.010** *	2.130	0.783–5.820	0.138
Age	1.020	0.979–1.060	0.372	1.010	0.968–1.060	0.551
Presence of ulcer						
No	Reference			Reference		
Yes	3.020	1.130–8.050	**0.027** *	2.400	0.798–7.220	0.119
Presence of nodules						
No	Reference			Reference		
Yes	11.00	3.860–31.70	**< 0.001** *	6.820	2.150–21.70	**0.001** *
NLR						
NLR < 3.03	Reference			Reference		
NLR ≥ 3.03	5.680	2.190–14.70	**< 0.001** *	4.260	1.430–12.70	**0.009** *

Abbreviations: 95% CI, 95% confidence interval; EMPD, extramammary Paget's disease; NLR, neutrophil‐to‐lymphocyte ratio.

* Bold letters indicate stastically significant differences: *p* < 0.05.

In univariate analysis, sex (male, odds ratio [OR]: 3.110, *p* = 0.010), presence of ulcers (OR: 3.020, *p* = 0.027), presence of nodules (OR: 11.00, *p* < 0.001), and NLR (≥ 3.03, OR: 5.680, *p* < 0.001) were significantly associated with DI of the primary lesion. In the multivariate analysis, presence of nodules (OR: 6.820, *p* = 0.001) and NLR (≥ 3.03, OR: 4.260, *p* = 0.009) were identified as independent predictive factors for DI of the primary lesion. In this study, there were 83 patients with NLR < 3.03 and no nodules, of whom 70 (84.3%) had IE lesions or MI. Conversely, 38 patients had either NLR ≥ 3.03 or a nodule at the primary site, of which 23 (60.5%) had DI.

### 3.4 Univariate and Multivariate Analyses of Potential Predictors of the SLN Metastases

The most sensitive and specific cutoff value of the NLR for predicting SLN metastasis was determined to be 2.87 using the ROC curve.

In univariate analysis, sex (male, OR: 3.680, *p* = 0.016), presence of nodules (OR: 12.30, *p* < 0.001), and NLR (≥ 2.87, OR: 6.870, *p* < 0.001) were significantly associated with SLN metastasis. In the multivariate analysis, the presence of nodules (OR: 8.460, *p* < 0.001) and NLR (≥ 2.87, OR: 3.870, *p* = 0.016) were identified as independent predictive factors for SLN metastasis (Table 4).

**TABLE 4:** Multiple logistic regression analysis of predictors of SLN metastasis in patients with EMPD.

Variable	Univariate analysis			Multivariate analysis		
	Odds ratio	95% CI	*p*	Odds ratio	95% CI	*p*
Sex						
Female	Reference			Reference		
Male	3.680	1.280–10.60	**0.016** *	2.470	0.696–8.74	0.162
Age	0.994	0.952–1.040	0.775	0.969	0.919–1.020	0.236
Presence of ulcer						
No	Reference			Reference		
Yes	2.750	0.871–8.680	0.085	1.320	0.355–4.90	0.670
Presence of nodules						
No	Reference			Reference		
Yes	12.30	4.320–35.00	**< 0.001** *	8.460	2.510–28.50	**< 0.001** *
NLR						
NLR < 2.87	Reference			Reference		
NLR ≥ 2.87	6.870	2.620–18.00	**< 0.001** *	3.870	1.280–11.70	**0.016** *

Abbreviations: 95% CI, 95% confidence interval; EMPD, extramammary Paget's disease; NLR, neutrophil‐to‐lymphocyte ratio; SLN, sentinel lymph node.

* Bold letters indicate statistically significant: *p* < 0.05.

### 3.5 OS by the Invasion Level of the Primary Tumor and SLN Metastasis

**FIGURE 1:**
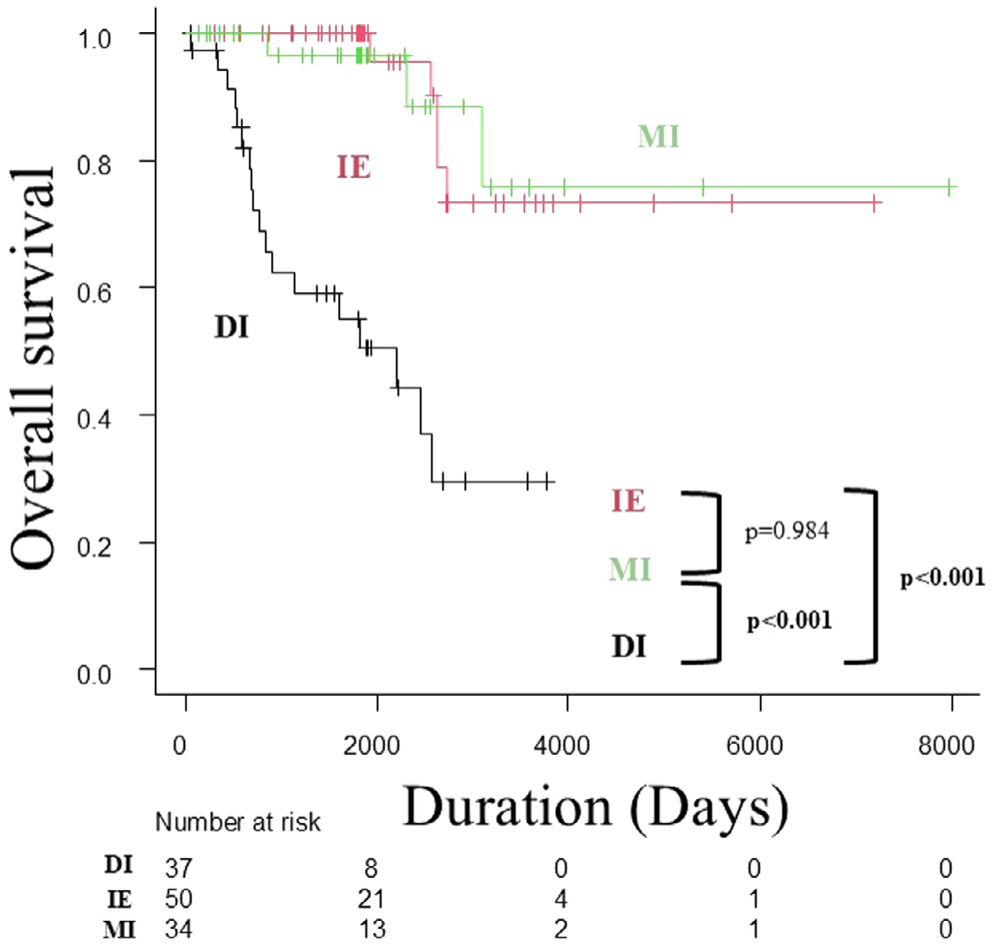
Kaplan–Meier analysis of overall survival by the invasion level of the primary tumor in patients with EMPD without clinical lymph node enlargement. There is no difference in the OS between the IE and MI groups; however, the DI group has a significantly worse prognosis than the other two groups (median OS: not reached [IE] vs. not reached [MI] vs. 73.0 months [DI], log‐rank test, *p* < 0.001). DI, deep invasion into/beyond the reticular dermis; EMPD, extramammary Paget's disease; IE, intraepidermal; MI, microinvasion into the papillary dermis; OS, overall survival.

**FIGURE 2:**
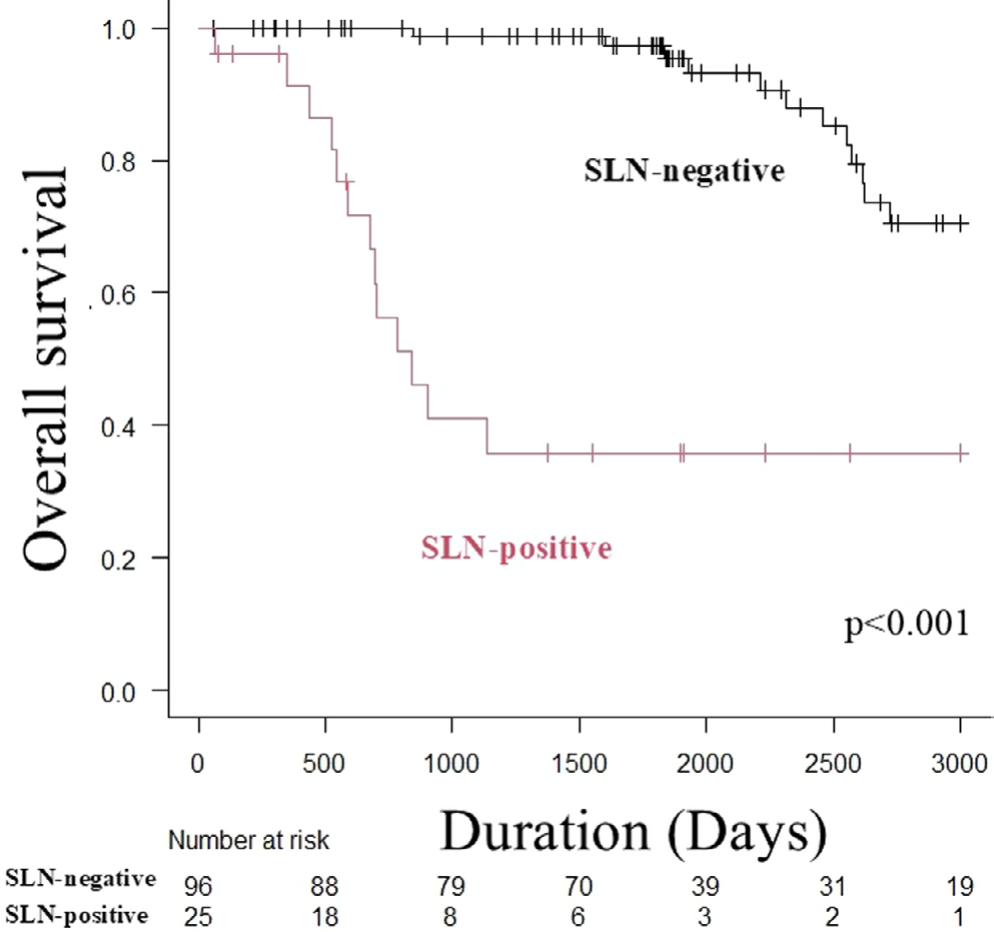
Kaplan–Meier analysis of overall survival in the SLN‐positive and negative groups of patients with EMPD without clinical lymph node enlargement. The SLN‐positive group has a significantly worse OS than the negative group (median OS: 27.6 months vs. not reached, log‐rank test, *p* < 0.001). EMPD, extramammary Paget's disease; OS, overall survival; SLN, sentinel lymph node.

**FIGURE 3:**
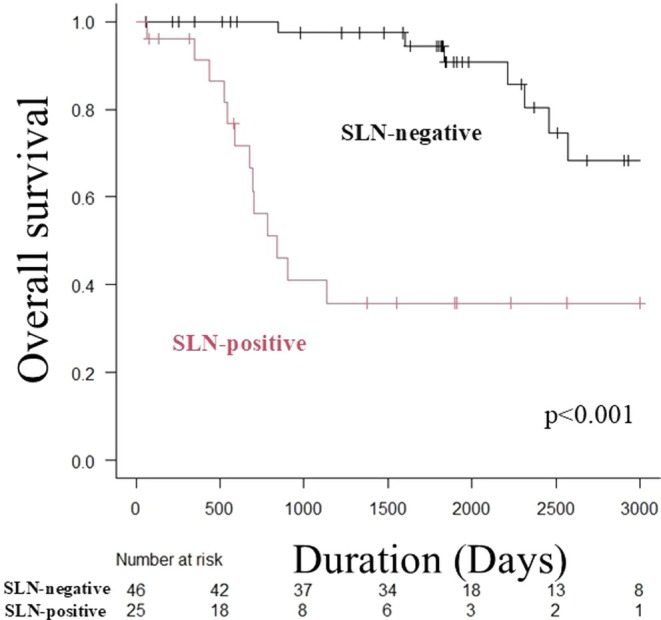
Kaplan–Meier analysis of overall survival in the SLN‐positive and SLN‐negative groups of patients with EMPD without clinical lymph node enlargement whose primary tumors are of MI and DI type. The SLN‐positive group has a significantly worse OS than the negative group (median OS: 27.6 months vs. not reached, log‐rank test, *p* < 0.001). DI, deep invasion into/beyond the reticular dermis; EMPD, extramammary Paget's disease; MI, microinvasion into the papillary dermis; OS, overall survival; SLN, sentinel lymph node.

There was no difference in OS between the IE and MI groups; however, the DI group had a significantly worse prognosis than the other two groups (Figure 1) (median OS: not reached [IE] vs. not reached [MI] vs. 73.0 months [DI], log‐rank test, *p* < 0.001). OS was significantly worse in the SLN‐positive group than in the negative group (median OS: 27.6 months vs. not reached, log‐rank test, *p* < 0.001) (Figure 2). Similar to the overall trend, when OS was compared by SLN metastasis in the MI and DI groups only, it was significantly worse in the SLN‐positive group (median OS: 27.6 months vs. not reached, log‐rank test, *p* < 0.001) (Figure 3).

## 4 Discussion

In this study, we examined the clinical features and biomarkers that predict the invasion level of the primary tumor and SLN metastasis in patients with EMPD of the genital area without enlarged lymph nodes. As in previous studies [3, 4, 5], no patient developed SLN metastasis when the primary tumor was IE (Table 2), indicating that SLNB is an unnecessary intervention when the primary tumor does not have dermal invasion. In addition, the SLN metastasis rate in the MI group was low at 5.9%, and there was no difference in prognosis compared with the IE group (Figure 1). Furthermore, a previous study reported a good prognosis in both the IE and MI groups who did not undergo SLNB [6]. Considering these facts, routine SLNB is considered excessive both in the IE and MI groups. However, considering the high metastasis rate, SLNB is important in patients with DI. Few retrospective cohort studies have investigated the predictive factors associated with primary dermal invasion. Therefore, we first examined the predictors of DI of the primary lesion and found that nodules at the primary site and preoperative NLR were independent predictors. In fact, the majority of patients (84.3%) with a low NLR (NLR < 3.03) and without nodules at the primary site had IE lesions or MI in this study. Therefore, we believe that in such cases, it would be a good option not to perform SLNB from the beginning but to resect the primary tumor first. This option is particularly recommended when primary resection can be performed under local anesthesia to avoid the risk of multiple general anesthesia sessions. Conversely, given that approximately 25% of patients were upstaged to DI after primary resection, even if a previous partial biopsy showed an IE lesion, it is advisable to perform primary resection and SLNB simultaneously in the presence of a high NLR or nodule.

We then examined the predictors of SLN metastasis in EMPD of the genital area without clinical lymph node enlargement and found that nodules at the primary site and NLR were independent predictors. Fujisawa et al. reported 107 patients with invasive EMPD (including MI and DI, excluding IE lesions) without enlarged lymph nodes who underwent SLNB [7]. A previous study reported that the SLN‐positive group had significantly more males (94% vs. 65%, *p* = 0.021), nodules (63% vs. 25%, *p* = 0.0029), DI (81% vs. 36%, *p* = 0.0008), and lymphovascular invasion at the primary site (44% vs. 5%, *p* < 0.0001). Multivariate analysis revealed that only DI (OR: 5.8, *p* = 0.04) and lymphovascular invasion (OR: 18.0, *p* = 0.0023) were independent predictors of SLN metastasis. These results were similar to those of the present study in that the invasion of the primary tumor affected SLN metastasis. However, with respect to OS, the previous study reported no significant difference in OS by the presence or absence of SLN metastasis (*p* = 0.69). However, Ogata et al. reported that, in invasive EMPD, the SLN‐positive group had a significantly worse 5‐year survival rate than the negative group (24% vs. 100%, *p* = 0.0001) [3]. In this study, the SLN‐positive group had a significantly poorer prognosis in the subgroup analysis (Figure 3), and we believe that SLNs are significant prognostic factors for invasive EMPD.

Regarding the SLN metastasis of EMPD and nodules at the primary site, in addition to a study by Fujisawa et al. [7], Hatta et al. reported that the presence of nodules at the primary site was not associated with SLN metastasis [5]. However, the study included only 13 patients and may not have been adequately examined statistically. As for OS and nodules at the primary site, Hatta et al. [8] reported that nodules were a poor prognostic factor in a univariate analysis of 76 patients with EMPD (*p* < 0.001), but not an independent poor prognostic factor in a multivariate analysis (*p* = 0.75). Additional research is required to determine the relationship between nodules at the primary site and tumor progression in EMPD.

In this study, no significant correlation was found between the primary tumor size and tumor invasion. Several studies have been conducted on the association between the size of the primary tumor and prognosis in EMPD, with some reporting that the larger the primary tumor, the more likely it is to metastasize to the lymph nodes [9] and the worse the prognosis [10], while others reported that there is no correlation between the dermal invasion [9], metastasis [4, 7, 11], and prognosis [11], and no consensus has been reached. One possible reason for this is that the measurement standards for size such as the longest diameter, product of the longest and shortest diameters, and area differ in each study. Another possible reason is the inaccurate measurement of the lesion size due to the unclear borders, the three‐dimensional structure of the genital area, and the elasticity of the scrotal skin. Further verification and discussion are necessary after standardizing the methods for measuring the size.

Regarding ulcers, Yasui et al. [10] reported that EMPD with erosion had a significantly worse prognosis than that without erosion (*p* < 0.001). In the present study, univariate analysis also showed that ulcers were significantly associated with DI (*p* = 0.027) and tended to increase SLN metastasis (*p* = 0.085). A multivariate analysis was not performed in the previous study; however, ulcers were not an independent predictor in the present study. Although ulcerative lesions, together with nodules, appear late in EMPD [12] and may be a poor prognostic factor, this study included clinically determined ulcers, and lesions that were not true ulcers or very small and shallow erosions might have influenced the results. To examine whether ulcers are truly a prognostic predictor, additional studies are required on ulcers that are pathologically confirmed to be deeper than a certain level.

There are two previous studies from Japan regarding the prognostic value of NLR for EMPD. Ebata et al. analyzed 137 patients with EMPD who underwent SLNB and reported that the high NLR group (NLR > 3) had significantly more SLN metastases than the low NLR group (NLR ≤ 3) (23% vs. 8%, *p* = 0.245) [13]. They also reported that the NLR was an independent predictor of SLN metastasis (NLR > 3, OR: 6.48, *p* = 0.014). Maeda et al. also analyzed 109 patients with EMPD and reported that the metastatic EMPD group had significantly higher NLR than the non‐metastatic EMPD group (mean NLR: 3.39 vs. 2.53, *p* = 0.005) and the high NLR group (NLR > 3) had significantly worse OS than the low NLR group (NLR ≤ 3) (*p* = 0.003) [14]. Based on the results of previous studies and this study, NLR may be predictive of the invasion level of the primary tumor and progression to the SLN and systemic organs in patients with EMPD.

We performed one of the largest retrospective cohort analyses of patients with EMPD of the genital area without clinical lymphadenopathy. We were then able to propose predictors of primary site invasiveness and SLN metastasis and the usefulness of SLNB as a predictor of OS. Nevertheless, this study has several limitations. First, it was a retrospective cohort study, which may have introduced a selection bias. Second, this study was conducted at a single institution and had a small sample size. Third, the NLR may be affected by diseases and patient conditions other than EMPD.

In conclusion, routine SLNB may be useful in predicting patient prognosis in patients with EMPD of the genital area without clinical lymph node enlargement. However, the likelihood of SLN metastasis is extremely low when the primary site has an IE lesion or MI. It may be reasonable to proactively recommend SLNB, particularly in cases with a confirmed DI lesion, the presence of nodules, or elevated NLR.

## Ethics Statement

Approval of the research protocol by an Institutional Review Board: Reviewed and approved by the Ethics Committee of Shizuoka Cancer Center (2024/9/19): approval #J2024‐234.

## Consent

The authors have nothing to report.

## Conflicts of Interest

The authors declare no conflicts of interest.

## Data Availability

Research data are not shared.

## References

[1] N. Kibbi , J. L. Owen , B. Worley , et al., “Evidence‐Based Clinical Practice Guidelines for Extramammary Paget Disease,” JAMA Oncology 8, no. 4 (2022): 618–628, 10.1001/jamaoncol.2021.7148. 35050310

[2] S. Matsushita , I. Kajihara , K. Tsutsui , et al., “Skin Cancer Clinical Practice Guidelines, 4th Edition Guideline for the Treatment of Extra‐Mammary Paget's Disease 2025,” Japanese Journal of Dermatology 135, no. 1 (2025): 1–36, 10.14924/dermatol.135.1.

[3] D. Ogata , Y. Kiyohara , S. Yoshikawa , and T. Tsuchida , “Usefulness of Sentinel Lymph Node Biopsy for Prognostic Prediction in Extramammary Paget's Disease,” European Journal of Dermatology 3, no. 26 (2016): 254–259, 10.1684/ejd.2016.2744. 26985569

[4] Y. Nakamura , Y. Fujisawa , M. Ishikawa , et al., “Usefulness of Sentinel Lymph Node Biopsy for Extramammary Paget Disease,” British Journal of Dermatology 4, no. 167 (2012): 954–956, 10.1111/j.1365-2133.2012.11017. 22533523

[5] N. Hatta , R. Morita , M. Yamada , et al., “Sentinel Lymph Node Biopsy in Patients With Extramammary Paget's Disease,” Dermatologic Surgery 30, no. 10 (2004): 1329–1334, 10.1111/j.1524-4725.2004.30377. 15458530

[6] M. van der Linden , M. Oonk MHM , H. C. van Doorn , et al., “Vulvar Paget Disease: A National Retrospective Cohort Study,” Journal of the American Academy of Dermatology 81, no. 4 (2019): 956–962, 10.1016/j.jaad.2018.11.016. 30458205

[7] Y. Fujisawa , K. Yoshino , Y. Kiyohara , et al., “The Role of Sentinel Lymph Node Biopsy in the Management of Invasive Extramammary Paget's Disease: Multi‐Center, Retrospective Study of 151 Patients,” Journal of Dermatological Science 79, no. 1 (2015): 38–42, 10.1016/j.jdermsci.2015.03.014. 25944505

[8] N. Hatta , M. Yamada , T. Hirano , A. Fujimoto , and R. Morita , “Extramammary Paget's Disease: Treatment, Prognostic Factors and Outcome in 76 Patients,” British Journal of Dermatology 158, no. 2 (2008): 313–318, 10.1111/j.1365-2133.2007.08314. 18028492

[9] Z. Kang , Q. Zhang , Q. Zhang , et al., “Clinical and Pathological Characteristics of Extramammary Paget's Disease: Report of 246 Chinese Male Patients,” International Journal of Clinical and Experimental Pathology 10, no. 8 (2015): 13233–13240. PMC4680468 26722523

[10] Y. Yasui , H. Kato , S. Kano , et al., “Impact of Lesion Location and Pattern on the Prognosis of Genital Extramammary Paget Disease: A Retrospective Study,” British Journal of Dermatology 192, no. 2 (2025): 365–367, 10.1093/bjd/ljae380. 39376022

[11] H. Escolà , B. Llombart , A. Escolà‐Rodríguez , et al., “Therapeutic Outcomes and Survival Analysis of Extramammary Paget's Disease: A Multicentre Retrospective Study of 249 Patients,” Journal of the American Academy of Dermatology 90, no. 1 (2024): 66–73, 10.1016/j.jaad.2023.08.088. 37704106

[12] S. Ishizuki and Y. Nakamura , “Extramammary Paget's Disease: Diagnosis, Pathogenesis, and Treatment With Focus on Recent Developments,” Current Oncology 4, no. 28 (2021): 2969–2986, 10.3390/curroncol28040260. PMC8395499 34436026

[13] A. Ebata , T. Taki , S. Mori , et al., “Neutrophil/Lymphocyte Ratio as a Predictor of Lymph Node Metastasis in Extramammary Paget Disease: A Retrospective Study,” Journal of the American Academy of Dermatology 85, no. 4 (2021): 1023–1025, 10.1016/j.jaad.2020.12.087. 33482256

[14] T. Maeda , J. Uehara , R. Toyoshima , T. Nakagawa , and K. Yoshino , “Neutrophil‐to‐Lymphocyte Ratio is a Potential Prognostic Biomarker for Extramammary Paget Disease: A Retrospective Study,” Journal of Dermatology 49, no. 11 (2022): 1188–1192, 10.1111/1346-8138.16533. 35903974

